# A System-Level Self-Calibration Method for Installation Errors in A Dual-Axis Rotational Inertial Navigation System

**DOI:** 10.3390/s19184005

**Published:** 2019-09-16

**Authors:** Shiyu Bai, Jizhou Lai, Pin Lyu, Xiaowei Xu, Ming Liu, Kai Huang

**Affiliations:** 1 Navigation Research Center, Nanjing University of Aeronautics and Astronautics, Nanjing 210000, China; 2 Overall Design Institute, Hubei Academy of Aerospace Technology, Wuhan 430000, China; 3 Shanxi Baocheng Aviation Instrument Corporation, Baoji 721000, China

**Keywords:** installation errors, system-level, self-calibration, observability analysis

## Abstract

In a dual-axis rotational inertial navigation system (RINS), there are two kinds of installation errors, nonorthogonal installation errors of inertial sensors, and installation errors between the inertial measurement unit (IMU) and rotation axes. Traditionally, these two errors are not considered simultaneously. Thus, they are calibrated separately by different estimation algorithms and rotation schemes. In this paper, a system-level self-calibration method for installation errors of a dual-axis RINS is proposed. Based on the Kalman filter, the measurement model is reestablished to ensure that all installation errors can be estimated together. First, the relationship between the initial attitude and subsequent attitude of IMU during rotation is used as a constraint to estimate nonorthogonal installation errors of accelerometers, and installation errors between the IMU and rotation axes. Then, the angular rate of the rotation mechanism is used as a reference to estimate nonorthogonal installation errors of the gyros. The rotation scheme of the IMU is designed to make all installation errors observable, and the observability of the system is analyzed based on the piecewise constant system method. Simulation and laboratory experiment results suggest that installation errors can be effectively estimated by the proposed method, thereby avoiding the complex separating process.

## 1. Introduction

The errors of inertial sensors are the major cause which influences the accuracy of an inertial navigation system (INS). Such errors can be decreased by enhancing the accuracy of inertial sensors. However, the cost and volume of the system can be heavily increased. Instead of using costly inertial sensors, the performance of the INS can be improved through rotary modulation (RM). The constant errors of inertial sensors can be compensated by the rotating inertial measurement unit (IMU) periodically [1,2]. Hence, the rotational INS (RINS) has attracted increasing attention in recent years. For example, the RM technique was applied to enhance the performance of the INS in an autonomous underwater vehicle [3]. A fiber optic gyros (FOG) single-axis RINS was combined with an odometer to improve the positioning and orientation accuracy of a land vehicle [4]. To further improve the performance of the RINS, an improved 8-position rotation scheme and a novel 16-position rotation scheme were proposed for the optical gyro-based RINS [1]. Meanwhile, error propagation characteristics and rotation scheme designing were analyzed [5]. The effect of RINS’s main error source, including constant drift of gyro and zero bias of accelerometer under rotation conditions, was analyzed [6]. The compensation effects of gyros’ stochastic errors were analyzed, and the specific formulae were derived [7].

The accuracy of RINS is influenced by many error parameters [8], among which the installation errors are of key importance. In RINS, installation errors consist of two parts, one is caused by nonorthogonal mounting of inertial sensors. The other is caused by misalignments between IMU axes and rotation axes. Conventionally, researchers have only focused on the calibration of either of these installation errors and designed corresponding calibration algorithms and schemes to estimate one type of error. Following this method makes it complicated to obtain all installation errors because different estimation algorithms and rotation schemes need to be adopted in turn. For the first type of installation errors, one study used velocity measurement of the navigation system to implement calibration [9]. Velocity and position errors were used as a measurement to estimate installation errors and accelerometer nonlinearity errors by applying the Kalman filter [10]. Another study utilized attitude and velocity errors to identify installation errors by applying an extended Kalman filter [11]. The relationship between navigation errors and IMU errors was established using an error model of navigation solution and initial alignment, and then the installation errors were estimated through a total least squares method [12]. The above calibration methods do not consider the effect of installation errors between the IMU and rotation axes, which affects the accuracy of attitude. For the second type of installation error, one study utilized the sensitivity of the gyroscope to the residual rotation, which is caused by installation errors. The errors could be estimated by applying a thin-shell method [13]. Another study used specific force to estimate installation errors through singular value decomposition [14]. The attitude difference of two RINSs was utilized to estimate installation errors via the Kalman filter [15]. Although the above references discuss the calibration of installation errors between the IMU and rotation axes, their methods still cannot obtain all installation errors.

To ensure all error parameters are observable, a specific rotation scheme needs to be designed to implement error excitation in all states. The rotation scheme design principles for errors calibration in RINS was discussed [10]. A self-calibration scheme, which included three separate steps to reduce the correlations between error parameters in tri-axis RINS, was designed [16]. Another study designed a self-calibration scheme for the gimbaled IMU, in which the multi-position and continuous rotation steps were processed alternately, the dynamic errors were suppressed, and the excitation was augmented [17].

In this paper, a system-level self-calibration method for two types of installation errors of a dual-axis RINS is proposed to avoid the complicated separating calibration process. Existing models and algorithms are only applicable for the calibration of either of these two installation errors. Thus, different estimation algorithms must be adopted in turn to obtain all errors. The major breakthrough is that all installation errors in the dual-axis RINS can be calibrated using the proposed system-level method. The point of difference to previous researches is that these two installation errors are considered simultaneously. Based on the Kalman filter, the measurement model is reestablished to ensure that all errors can be estimated together. First, the relationship between initial attitude and sequent attitude calculated from specific forces is used to establish the observation equation, and nonorthogonal installation errors of accelerometers and installation errors between IMU axes and rotation axes can be estimated. Then, the estimated outputs of the first step are employed in the formulation of the relationship between gyroscope and angular rate of rotational mechanism, and nonorthogonal installation errors of gyros can be obtained. The rotation scheme of the IMU is designed to make all installation errors observable, and the observability of the system is analyzed based on the piecewise constant system (PWCS) method. Finally, the proposed self-calibration method is validated through simulation and experiment.

## 2. Analysis of the IMU Error Model

### 2.1. Coordinate System and Transformation

A few coordinates need to be established to describe spatial relationships among different parameter variables.

#### 2.1.1. IMU Frame

**Figure 1:**
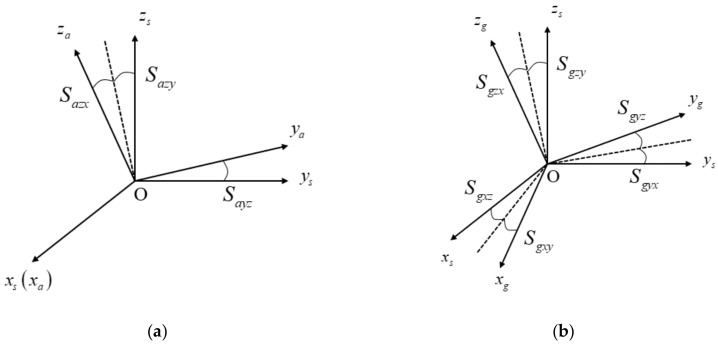
Definition of the inertial measurement unit (IMU) frame, accelerometers frame, and gyro frame. (**a**) IMU frame and accelerometer frame; (**b**) IMU frame and gyro frame.

The outputs of accelerometers and gyroscopes are measured in their respective coordinates, denoted by a-frame and g-frame. The three axes of a-frame are denoted as $x_{a}$, $y_{a}$, $z_{a}$, and the three axes of g-frame are denoted by $x_{g}$, $y_{g}$, $z_{g}$. Because a-frame and g-frame are both nonorthogonal frames, the outputs of inertial sensors need to be transformed to an orthogonal coordinate, called the IMU frame, denoted by s-frame. The three axes of s-frame are denoted by $x_{s}$, $y_{s}$, $z_{s}$. The s-frame is defined as follows [10]: $x_{s}$ coincides with $x_{a}$,$\;y_{s}$ is in the $x_{a}$, $y_{a}$ plane, while $z_{s}$ forms a right-handed orthogonal frame with $x_{s}$ and $y_{s}$. The spatial relationships among a-frame, g-frame, and s-frame are shown in Figure 1.

The transformation matrices from a-frame and g-frame to s-frame are expressed as follows:

(1) $$\begin{array}{l} C_{a}^{s}=\left[\begin{array}{ccc} 1 & 0 & 0\\ S_{ayz} & 1 & 0\\ -S_{azy} & S_{azx} & 1 \end{array}\right]\\ C_{g}^{s}=\left[\begin{array}{ccc} 1 & -S_{gxz} & S_{gxy}\\ S_{gyz} & 1 & -S_{gyx}\\ -S_{gzy} & S_{gzx} & 1 \end{array}\right] \end{array},$$

where $S_{ayz}$, $S_{azx}$, and $S_{azy}$ are nonorthogonal installation errors of accelerometers and $S_{gxy}$, $S_{gxz}$, $S_{gyx}$, $S_{gyz}$, $S_{gzx}$, and $S_{gzy}$ are nonorthogonal installation errors of gyros.

In the process of rotation, s-frame rotates along with inertial sensors. A new frame needs to be introduced to describe the s-frame in the initial state, denoted by s0-frame.

#### 2.1.2. Rotational Mechanism Frame

**Figure 2:**
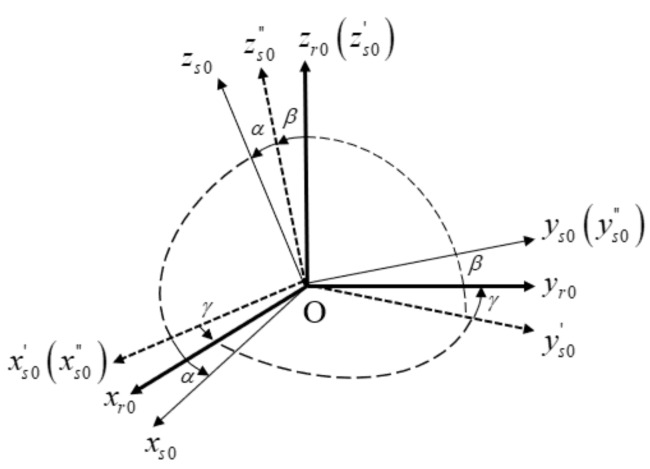
Definition of IMU frame and rotational mechanism frame in the initial state.

The rotational mechanism frame needs to be established to describe the spatial relationship between IMU axes and rotation axes. Aimed at the dual-axis RINS, in the initial state, the rotational mechanism frame is denoted by r0-frame; its three axes are defined by $x_{r0}$, $y_{r0}$, $z_{r0}$. Assuming that the inner axis and outer axis are perpendicular, $x_{r0}$ coincides with the outer axis, $z_{r0}$ coincides with the inner axis, while $y_{r0}$ forms a right-handed orthogonal frame with $x_{r0}$ and $z_{r0}$. In the initial state, r0-frame and s0-frame do not coincide with each other due to installation errors. There is a transformation between r0-frame and s0-frame, which is shown in Figure 2.

Considering that installation errors are small, the transformation matrix between r0-frame and s0-frame is represented as

(2) $$C_{r0}^{s0}=\left[\begin{array}{ccc} 1 & -\gamma & \alpha \\ \gamma & 1 & -\beta \\ -\alpha & \beta & 1 \end{array}\right],$$

where α, β, and γ are installation errors between IMU axes and rotation axes.

Meanwhile, a new frame, denoted by r-frame is used to describe the rotational mechanism frame in the process of rotation. The transformation matrix from r0-frame to r-frame can be represented as follows:

(3) $$\begin{array}{l} C_{r0}^{r}=R_{x}\left(A_{x}\right)R_{z}\left(A_{z}\right)\\ =\left[\begin{array}{ccc} 1 & 0 & 0\\ 0 & c\left(A_{x}\right) & s\left(A_{x}\right)\\ 0 & -s\left(A_{x}\right) & c\left(A_{x}\right) \end{array}\right]\left[\begin{array}{ccc} c\left(A_{z}\right) & s\left(A_{z}\right) & 0\\ -s\left(A_{z}\right) & c\left(A_{z}\right) & 0\\ 0 & 0 & 1 \end{array}\right], \end{array}$$

where $A_{x}$ and $A_{z}$ are the rotation angle of the outer and inner axis, respectively, c and s represent the trigonometric operator cos and sin, respectively. Meanwhile, the transformation matrix between s-frame and r-frame satisfies Equation (2).

#### 2.1.3. Body Frame

The body frame is fixed on the vehicle, denoted by b-frame. The three axes of b-frame are defined as $x_{b}$, $y_{b}$, $z_{b}$. $x_{b}$ axis points rightward, $y_{b}$ axis points forward, and $z_{b}$ points upward. In this paper, b-frame coincides with s0-frame.

#### 2.1.4. Navigation Frame

The navigation frame is denoted by n-frame, its three axes are defined as $x_{n}$, $y_{n}$, $z_{n}$. $x_{n}$ axis points east, $y_{n}$ axis point north, and $z_{n}$ axis points up.

### 2.2. Error Modeling of Inertial Sensors

Based on spatial relationship from Equation (1), the model of inertial sensors can be expressed as follows:

(4) $$\begin{array}{ll} f^{s} & =C_{a}^{s}f^{a}\\ & =\left[\begin{array}{ccc} 1 & 0 & 0\\ S_{ayz} & 1 & 0\\ -S_{azy} & S_{azx} & 1 \end{array}\right]\left[\begin{array}{c} f_{x}^{a}\\ f_{y}^{a}\\ f_{z}^{a} \end{array}\right]\\ & =\left[\begin{array}{c} f_{x}^{a}\\ S_{ayz}f_{x}^{a}+f_{y}^{a}\\ -S_{azy}f_{x}^{a}+S_{azx}f_{y}^{a}+f_{z}^{a} \end{array}\right] \end{array},$$

where $f^{a}$ is the output of accelerometers measured in a-frame, $f^{s}$ is the output of accelerometers expressed in s-frame.

(5) $$\begin{array}{ll} \omega^{s} & =C_{g}^{s}\omega^{g}\\ & =\left[\begin{array}{ccc} 1 & -S_{gxz} & S_{gxy}\\ S_{gyz} & 1 & -S_{gyx}\\ -S_{gzy} & S_{gzx} & 1 \end{array}\right]\left[\begin{array}{c} \omega_{x}^{g}\\ \omega_{y}^{g}\\ \omega_{z}^{g} \end{array}\right]\\ & =\left[\begin{array}{c} \omega_{x}^{g}-S_{gxz}\omega_{y}^{g}+S_{gxy}\omega_{z}^{g}\\ S_{gyz}\omega_{x}^{g}+\omega_{y}^{g}-S_{gyx}\omega_{z}^{g}\\ -S_{gzy}\omega_{x}^{g}+S_{gzx}\omega_{y}^{g}+\omega_{z}^{g} \end{array}\right] \end{array},$$

where $\omega^{g}$ is the output of gyros measured in g-frame, $\omega^{s}$ is the output of gyros expressed in s-frame.

## 3. A Self-Calibration Method Based on the Kalman Filter

**Figure 3:**
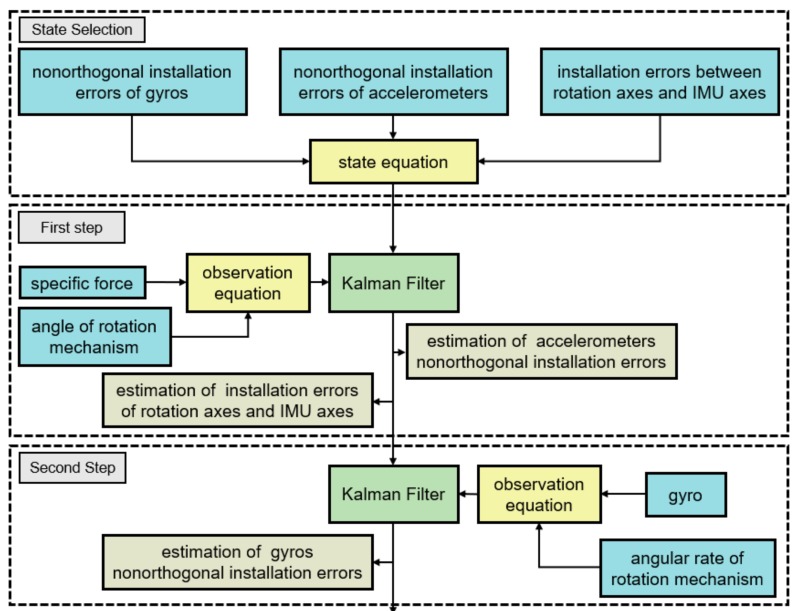
Structure of system-level self-calibration method.

The structure of the self-calibration method is shown in Figure 3.

First, the vehicle attitude calculated from the rotation angle and IMU attitude should be the same as the IMU attitude in the initial state. This constraint can be used to establish the first observation equation. Specific forces are introduced to get IMU attitudes at each moment, and angles of the rotational mechanism are used to project IMU attitudes to b-frame. The nonorthogonal installation errors of accelerometers, and installation errors of IMU axes and rotation axes can be estimated through the first step. 

Second, the angular rate of rotational mechanism can be utilized as a reference to calibrate nonorthogonal installation errors of gyros. However, the angular rates of the earth also exist in the output of gyros and should be compensated. The estimation results of the first step, along with the angle of the rotational mechanism, can be used to project the angular rate of the earth from n-frame to s-frame. The second observation equation is established based on the relationship among outputs of gyros, angular rate of the earth and rotational mechanism, then the nonorthogonal installation errors of gyros can be estimated.

### 3.1. Design of the Kalman Filter

The states of the filter are installation errors, which are modeled as random constants, their dynamic models are expressed as follows:

(6) $$\begin{array}{l} X=[S_{gxy}\;S_{gxz}\;S_{gyx}\;S_{gyz}\;S_{gzx}\;S_{gzy}\;S_{ayz}\;S_{azx}\;S_{azy}\;\alpha \;\beta \;\gamma ]\\ X^{k}=FX^{k-1}+W^{k}=I_{12}X^{k-1}+W^{k} \end{array},$$

where $I_{12}$ is a 12-dimension identity matrix, $W^{k}$ is system noise.

Attitude calculated by projecting IMU attitude to b-frame equals to the IMU attitude in the initial state. Thus, this can be regarded as a constraint to construct an observation equation. Based on the attitude calculation process in RINS, the following equation can be obtained

(7) $$C_{n}^{b}=C_{s}^{b}C_{n}^{s}=C_{s}^{s0}C_{n}^{s}=C_{n}^{s0},$$

where $C_{n}^{b}$ is the attitude matrix of the vehicle, $C_{n}^{s}$ is the attitude matrix of the IMU during the rotation process, $C_{n}^{s0}$ is the attitude matrix of the IMU in the initial state, $C_{s}^{b}$ and $C_{s}^{s0}$ are the attitude matrices which can project the IMU attitude to b-frame.

Since there are installation errors between IMU axes and rotation axes, Equation (7) can be rewritten as

(8) $$\begin{array}{c} C_{n}^{s}=C_{s0}^{s}C_{n}^{s0}=C_{r}^{s}C_{r0}^{r}C_{s0}^{r0}C_{n}^{s0}\\ C_{n}^{s}=\left[\begin{array}{ccc} C_{11} & C_{12} & C_{13}\\ C_{21} & C_{22} & C_{23}\\ C_{31} & C_{32} & C_{33} \end{array}\right]\\ C_{r}^{s}=\left[\begin{array}{ccc} 1 & -\gamma & \alpha \\ \gamma & 1 & -\beta \\ -\alpha & \beta & 1 \end{array}\right]\\ C_{r0}^{r}=\left[\begin{array}{ccc} A_{11} & A_{12} & A_{13}\\ A_{21} & A_{22} & A_{23}\\ A_{31} & A_{32} & A_{33} \end{array}\right]\\ C_{s0}^{r0}=\left[\begin{array}{ccc} 1 & \gamma & -\alpha \\ -\gamma & 1 & \beta \\ \alpha & -\beta & 1 \end{array}\right]\\ C_{n}^{s0}=\left[\begin{array}{ccc} C_{11}^{0} & C_{12}^{0} & C_{13}^{0}\\ C_{21}^{0} & C_{22}^{0} & C_{23}^{0}\\ C_{31}^{0} & C_{32}^{0} & C_{33}^{0} \end{array}\right] \end{array},$$

where $C_{r}^{s}$ and $C_{s0}^{r0}$ are transfer matrices between the IMU and rotational mechanism, $C_{r0}^{r}$ is the transfer matrix calculated from the angle of the rotational mechanism. The third column of $C_{n}^{s}$ and $C_{n}^{s0}$ can be represented as follows by specific forces:

(9) $$\begin{array}{l} \left\{\begin{array}{l} C_{13}=-\sin\left(r\right)\cos\left(\theta \right)=\frac{f_{x}^{s}}{g}\\ C_{23}=\sin\left(\theta \right)=\frac{f_{y}^{s}}{g}\\ C_{33}=\cos\left(r\right)\cos\left(\theta \right)=\frac{f_{z}^{s}}{g} \end{array}\right.\\ \left\{\begin{array}{l} C_{13}^{0}=-\sin\left(r_{0}\right)\cos\left(\theta_{0}\right)=\frac{f_{x}^{s0}}{g}\\ C_{23}^{0}=\sin\left(\theta_{0}\right)=\frac{f_{y}^{s0}}{g}\\ C_{33}^{0}=\cos\left(r_{0}\right)\cos\left(\theta_{0}\right)=\frac{f_{z}^{s0}}{g} \end{array}\right. \end{array}$$

where $C_{13}$, $C_{23}$, $C_{33}$ are entries of $C_{n}^{s}$. $C_{13}^{0}$, $C_{23}^{0}$, $C_{33}^{0}$ are entries of $C_{n}^{s0}$. r and θ are roll and pitch of the IMU during the rotation process, $r_{0}$ and $\theta_{0}$ are roll and pitch of the IMU in the initial state, g is the gravity vector, $f^{s}$ and $f^{s0}$ are outputs of accelerometers expressed in the rotation process and initial state, respectively.

From Equations (8) and (9), Equation (10) is obtained as follows:

(10) $$\left[\begin{array}{c} f_{x}^{s}\\ f_{y}^{s}\\ f_{z}^{s} \end{array}\right]=C_{r}^{s}C_{r0}^{r}C_{s0}^{r0}\left[\begin{array}{c} f_{x}^{s0}\\ f_{y}^{s0}\\ f_{z}^{s0} \end{array}\right]$$

By substituting $f^{s}$ and $f^{s0}$ into Equation (10) with Equation (4), the second-order small quantity can be ignored, then the first observation equation at k can be represented as

(11) $$\begin{array}{c} Z_{1}^{k}=H_{1}X^{k}+V_{1}^{k}=\left[\begin{array}{cc} 0_{3\times 6} & \bar{H} \end{array}\right]X^{k}+V_{1}^{k}\\ Z_{1}^{k}=\left[\begin{array}{c} -f_{x}^{ak}+A_{11}f_{x}^{a0}+A_{12}f_{y}^{a0}+A_{13}f_{z}^{a0}\\ -f_{y}^{ak}+A_{21}f_{x}^{a0}+A_{22}f_{y}^{a0}+A_{23}f_{z}^{a0}\\ -f_{z}^{ak}+A_{31}f_{x}^{a0}+A_{32}f_{y}^{a0}+A_{33}f_{z}^{a0} \end{array}\right]\\ X^{k}={\left[\begin{array}{cccc} S_{gxy}^{k} & S_{gxz}^{k} & S_{gyx}^{k} & \begin{array}{cccc} S_{gyz}^{k} & S_{gzx}^{k} & S_{gzy}^{k} & \begin{array}{cccc} S_{ayz}^{k} & S_{azx}^{k} & S_{azy}^{k} & \begin{array}{ccc} \alpha^{k} & \beta^{k} & \gamma^{k} \end{array} \end{array} \end{array} \end{array}\right]}^{T}\\ {\bar{H}}_{11}=A_{12}f_{x}^{a0}\\ {\bar{H}}_{12}=A_{13}f_{y}^{a0}\\ {\bar{H}}_{13}=-A_{13}f_{x}^{a0}\\ {\bar{H}}_{14}=A_{31}f_{x}^{a0}+A_{13}f_{x}^{a0}+A_{32}f_{y}^{a0}-A_{11}f_{z}^{a0}+A_{33}f_{z}^{a0}\\ {\bar{H}}_{15}=A_{12}f_{z}^{a0}-A_{13}f_{y}^{a0}\\ {\bar{H}}_{16}=-A_{21}f_{x}^{a0}-A_{12}f_{x}^{a0}+A_{11}f_{y}^{a0}-A_{22}f_{z}^{a0}-A_{23}f_{z}^{a0}\\ {\bar{H}}_{21}=A_{22}f_{x}^{a0}-f_{x}^{a}\\ {\bar{H}}_{22}=A_{23}f_{y}^{a0}\\ {\bar{H}}_{23}=-A_{23}f_{x}^{a0}\\ {\bar{H}}_{24}=A_{23}f_{x}^{a0}-A_{21}f_{z}^{a0}\\ {\bar{H}}_{25}=-A_{31}f_{x}^{a0}-A_{32}f_{y}^{a0}-A_{23}f_{y}^{a0}+A_{22}f_{z}^{a0}-A_{33}f_{z}^{a0}\\ {\bar{H}}_{26}=A_{11}f_{x}^{a0}-A_{22}f_{x}^{a0}+A_{21}f_{y}^{a0}+A_{12}f_{y}^{a0}+A_{13}f_{z}^{a0}\\ {\bar{H}}_{31}=A_{32}f_{x}^{a0}\\ {\bar{H}}_{32}=A_{33}f_{y}^{a0}-f_{y}^{a}\\ {\bar{H}}_{33}=-A_{33}f_{x}^{a0}+f_{x}^{a}\\ {\bar{H}}_{34}=A_{33}f_{x}^{a0}-A_{11}f_{x}^{a0}-A_{12}f_{y}^{a0}-A_{31}f_{z}^{a0}-A_{13}f_{z}^{a0}\\ {\bar{H}}_{35}=A_{21}f_{x}^{a0}+A_{22}f_{y}^{a0}-A_{33}f_{y}^{a0}+A_{32}f_{z}^{a0}+A_{23}f_{z}^{a0}\\ {\bar{H}}_{36}=-A_{32}f_{x}^{a0}+A_{31}f_{y}^{a0} \end{array}$$

Equation (11) is based on the invariance of the vehicle’s attitude in a static situation. During the calibration process, the IMU rotates with the rotation mechanism while the vehicle is static. Therefore, the initial attitude of the IMU should equal to the vehicle attitude that can be obtained by projecting the subsequent attitude of the IMU during rotation to b-frame. Considering that the vehicle is in a static situation while the IMU rotates with the axes and rotation needs to stop at certain points during the calibration, the effect of a disturbance, such as moving or centripetal acceleration, can be ignored. Specific force is used to calculate the attitude of the IMU. Thus, the measurement z in Equation (11) includes specific force from accelerometers and angular data from the rotation mechanism.

In the rotation process, the outputs of gyros expressed in s-frame can be written as

(12) $$\begin{array}{l} \omega^{s}=\omega_{ie}^{s}+\omega_{r}^{s}=C_{n}^{s}\omega_{ie}^{n}+C_{r}^{s}\omega^{r}\\ =\left[\begin{array}{ccc} C_{11} & C_{12} & C_{13}\\ C_{21} & C_{22} & C_{23}\\ C_{31} & C_{32} & C_{33} \end{array}\right]\left[\begin{array}{c} 0\\ \omega_{ie}c\left(L\right)\\ \omega_{ie}s\left(L\right) \end{array}\right]+\left[\begin{array}{ccc} 1 & -\gamma & \alpha \\ \gamma & 1 & -\beta \\ -\alpha & \beta & 1 \end{array}\right]{\bar{\omega }}^{rota} \end{array},$$

where $\omega^{s}$ is the output of gyros in s-frame, $\omega_{ie}^{s}$ is the angular rate of the earth expressed in s-frame, $\omega_{r}^{s}$ and ${\bar{\omega }}^{rota}$ are angular rates of the rotational mechanism expressed in s-frame and r-frame, respectively. L is the latitude. The estimation results of the first step are utilized in Equation (12). First, the outputs of accelerometers in the initial state are recalculated via Equation (4) with the estimation of the nonorthogonal installation errors. A modified $C_{n}^{s0}$ is obtained. Then an estimation of installation errors between IMU axes and rotation axes along with angle of the rotational mechanism is used to calculate $C_{n}^{s}$ through Equation (8). $C_{n}^{s}$ is substituted into Equation (12) and the angular rate of the earth measured in s-frame can be obtained. Estimation of installation errors between IMU axes and rotation axes combined with the angular rate of the rotational mechanism is substituted into Equation (12) to get the residual rotation $\omega_{r}^{s}$. By substituting $\omega^{s}$ in Equation (12) with Equation (5), the second observation equation at k can be represented as

(13) $$\begin{array}{c} Z_{2}^{k}=H_{2}X^{k}+V_{2}^{k}=\left[\begin{array}{cc} \bar{H} & 0_{3\times 6} \end{array}\right]X^{k}+V_{2}^{k}\\ Z_{2}^{k}=\left[\begin{array}{c} C_{12}\omega_{ie}^{n}c\left(L\right)+C_{13}\omega_{ie}^{n}s\left(L\right)+{\bar{\omega }}_{x}^{rotak}-\gamma {\bar{\omega }}_{y}^{rotak}+\alpha {\bar{\omega }}_{z}^{rotak}-\omega_{isx}^{sk}\\ C_{22}\omega_{ie}^{n}c\left(L\right)+C_{23}\omega_{ie}^{n}s\left(L\right)+\gamma {\bar{\omega }}_{x}^{rotak}+\gamma {\bar{\omega }}_{y}^{rotak}-\beta {\bar{\omega }}_{z}^{rotak}-\omega_{isy}^{sk}\\ C_{32}\omega_{ie}^{n}c\left(L\right)+C_{33}\omega_{ie}^{n}s\left(L\right)-\alpha {\bar{\omega }}_{x}^{rotak}+\beta {\bar{\omega }}_{y}^{rotak}+\beta {\bar{\omega }}_{z}^{rotak}-\omega_{isz}^{sk} \end{array}\right]\\ X^{k}={\left[\begin{array}{cccc} S_{gxy}^{k} & S_{gxz}^{k} & S_{gyx}^{k} & \begin{array}{cccc} S_{gyz}^{k} & S_{gzx}^{k} & S_{gzy}^{k} & \begin{array}{cccc} S_{ayz}^{k} & S_{azx}^{k} & S_{azy}^{k} & \begin{array}{ccc} \alpha^{k} & \beta^{k} & \gamma^{k} \end{array} \end{array} \end{array} \end{array}\right]}^{T}\\ \bar{H}=\left[\begin{array}{cccccc} -\omega_{z}^{g} & \omega_{y}^{g} & 0 & 0 & 0 & 0\\ 0 & 0 & \omega_{z}^{g} & -\omega_{x}^{g} & 0 & 0\\ 0 & 0 & 0 & 0 & -\omega_{y}^{g} & \omega_{x}^{g} \end{array}\right] \end{array}$$

Equation (13) is based on that angular rate of the rotation mechanism and can be used as a high-precision reference. During the rotation, the output of gyros includes the angular rate of the earth and rotation mechanism and nonorthogonal installation error terms. The angular velocity of each axes can be directly obtained from the rotation mechanism. Thus, the measurement z in Equation (13) includes the angular rate from gyros, earth, and rotation mechanism.

### 3.2. Rotation Scheme Design and Observability Analysis

An appropriate rotation scheme should be designed to ensure all state variables observable. 

The rotation scheme refers to the design principles in study [10], which involve pointing upward and downward alternately for each accelerometer, and bidirectional rotation on each gyro. The specific rotation scheme goes as follows. The outer axis rotates bidirectionally first and stops every time the rotation angle is on a multiple of −90 degree. Second, the inner axis rotates to −90 degree. The outer axis rotates bidirectionally as in the last step. Finally, the inner axis rotates bidirectionally. Then, the first period of rotation is over, and the second repeats the above process. The angular velocity is set as 20 deg/s, the duration for every stopping point is 200 s. Equation (11) is used when the IMU stops during the first period of rotation, which is designed for avoiding the influence of centripetal acceleration. Equation (13) is used when the IMU rotates during the second period of rotation so that the gyro installation error can be activated.

**Figure 4:**
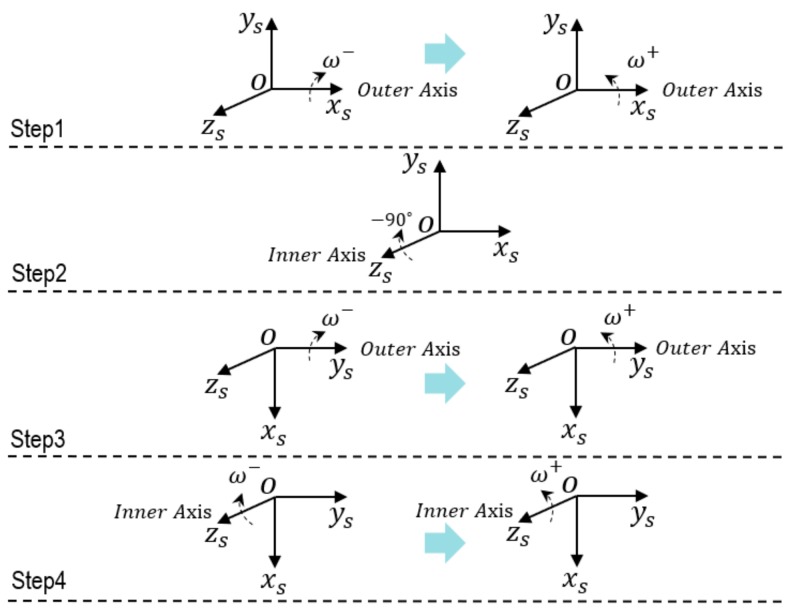
One period of rotation in the self-calibration scheme.

**Figure 5:**
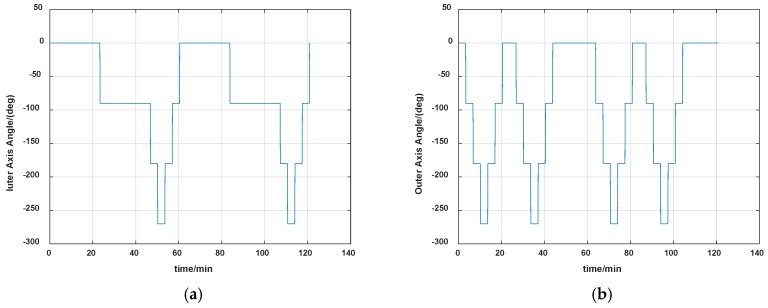
Angle changes of the inner and outer axis during the whole calibration. (**a**) Inner axis angle; (**b**) Outer axis angle.

One period of rotation for the installation error calibration is shown in Figure 4, and the angle changes of the inner and outer axis during the whole calibration scheme are shown in Figure 5.

#### 3.2.1. The Installation Errors’ Excitation of the First Period

1. Rotation along $x_{s}$ of the IMU

In the initial state, $x_{s}$ of the IMU nearly coincides with the outer axis of rotational mechanism and the IMU rotates along its *x_s_* axis when outer axis rotates. Then, Equation (13), as the rotation angle is on a multiple of −90 degree, can be approximated as

(14) $$\left\{\begin{array}{ll} f_{x}^{a}=g\gamma +g\alpha ,f_{y}^{a}=-gS_{azx} & A_{x}=-\frac{\pi }{2}\\ f_{x}^{a}=2g\gamma & A_{x}=-\pi \\ f_{x}^{a}=g\gamma -g\alpha ,f_{y}^{a}=gS_{azx} & A_{x}=-\frac{3\pi }{2} \end{array}\right..$$

It can be seen that α, γ, and $S_{azx}$ are activated when the IMU rotates along $x_{s}$ axis.

2. Rotation along $y_{s}$ of IMU

After the inner axis of rotational mechanism rotates to −90 degree, $y_{s}$ of the IMU nearly coincides with the outer axis, and the output of accelerometers at this moment approximates ${\left[\begin{array}{ccc} -g & 0 & 0 \end{array}\right]}^{T}$. When outer axis rotates, the IMU rotates along its $y_{s}$ axis, and Equation (13) can be approximated as

(15) $$\left\{\begin{array}{ll} f_{x}^{a}=gS_{azy},f_{y}^{a}=-gS_{ayz}-g\beta +g\gamma & A_{x}=-\frac{\pi }{2}\\ f_{y}^{a}=-2gS_{ayz}+2g\gamma ,f_{z}^{a}=0 & A_{x}=-\pi \\ f_{x}^{a}=-gS_{azy},f_{y}^{a}=-gS_{ayz}+g\beta +g\gamma & A_{x}=-\frac{3\pi }{2} \end{array}\right.$$

It can be seen that β, γ, $S_{ayz}$, and $S_{azy}$ are activated.

3. Rotation along $z_{s}$ of the IMU

After rotation along $z_{s}$ of the IMU, the output of the accelerometers still approximates ${\left[\begin{array}{ccc} -g & 0 & 0 \end{array}\right]}^{T}$. Due to the rotation of the inner axis in the second step, the inner axis cannot rotate −270 degrees. Thus, Equation (13) can be approximated as

(16) $$\left\{\begin{array}{ll} f_{x}^{a}=gS_{ayz},f_{z}^{a}=gS_{azx}+gS_{azy}-g\alpha -g\beta & A_{z}=-\frac{\pi }{2}\\ f_{z}^{a}=2gS_{azy}-2g\alpha & A_{z}=-\pi \end{array}\right.$$

It can be seen that α, β, $S_{ayz}$, $S_{azx}$, and $S_{azy}$ are activated.

#### 3.2.2. The Installation Errors’ Excitation of the Second Period

When the IMU rotates along the $z_{s}$ axis, the components of the angular rate caused by nonorthogonal installation errors can be expressed as

(17) $$\left\{\begin{array}{l} \omega_{g}^{x}=-S_{gxy}\omega_{s}^{z}\\ \omega_{g}^{y}=S_{gyx}\omega_{s}^{z} \end{array}\right..$$

It can be seen that $S_{gxy}$ and $S_{gyx}$ are activated. In the same way, $S_{gxz}$, $S_{gzx}$, $S_{gyz}$, and $S_{gzy}$ can be activated when the IMU rotates along the other two axes.

In this paper, the PWCS method is used to validate the observability of the system. When the time interval is small enough, the state matrix and observation matrix can be regarded as constant matrices in every time interval. The linear time-varying system becomes a linear time-invariant system in every time interval, which is the PWCS. In the ith time interval, the observable matrix of the dynamic system is

(18) $$Q_{i}={\left[H{\left(i\right)}^{T}\;{\left(H\left(i\right)A\left(i\right)\right)}^{T}\;{\left(H\left(i\right)A^{2}\left(i\right)\right)}^{T}\;\cdots \;{\left(H\left(i\right)A^{n-1}\left(i\right)\right)}^{T}\right]}^{T},$$

where $A\left(i\right)$ and $H\left(i\right)$ are the state matrix and the observation matrix in the ith time interval, n is the dimension of the state. The total observability matrix (TOM) of the dynamic system is

(19) $$Q_{TO}\left(r\right)=\left[\begin{array}{l} Q_{1}\\ Q_{2}e^{A_{1}\Delta_{1}}\\ Q_{3}e^{A_{2}\Delta_{2}}e^{A_{1}\Delta_{1}}\\ \cdots \\ Q_{r}e^{A_{r-1}\Delta_{r-1}}\cdots e^{A_{1}\Delta_{1}} \end{array}\right],$$

where ${∆}_{i}$ is the ith time interval, and the stripped observability matrix (SOM) of the dynamic system is

(20) $$Q_{SO}\left(r\right)={\left[Q_{1}^{T}\;Q_{2}^{T}\;Q_{3}^{T}\;\cdots \;Q_{r}^{T}\right]}^{T}.$$

**Figure 6:**
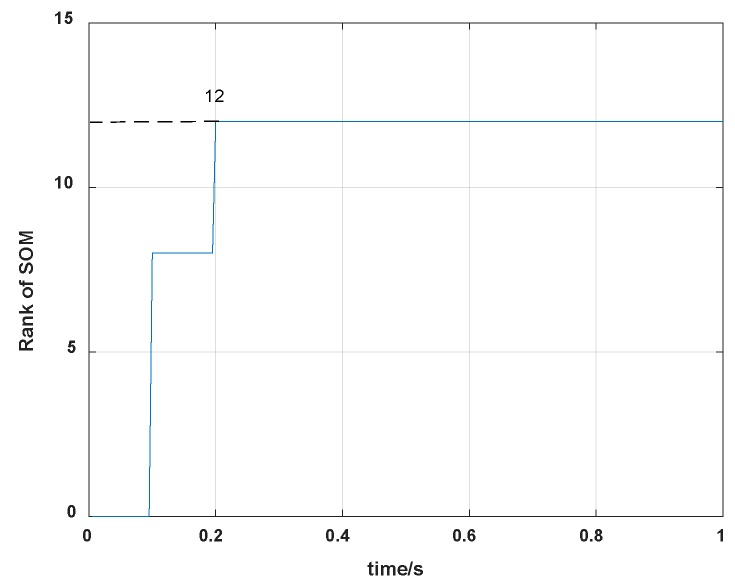
Rank change of the system.

When the rank of $Q_{SO}\left(r\right)$ is n, the system if fully observable, while the rank of $Q_{SO}\left(r\right)$ is less than n, the system is not fully observable. In our self-calibration scheme, ${∆}_{i}$ is set 0.005s, and the rank change of the system is shown in Figure 6.

From Figure 6, we can see that the dynamic system reaches full rank after 0.2 s, which is not the convergence time but indicates the system can be fully observable. Therefore, all state variables are observable in the proposed self-calibration scheme.

## 4. Simulation and Experiment

### 4.1. Simulation and Analysis

**Figure 7:**
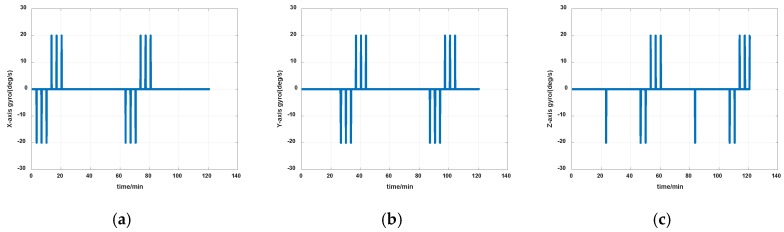
Measurement data of gyros in simulation (**a**) *x*-axis; (**b**) *y*-axis; (**c**) *z*-axis.

**Figure 8:**
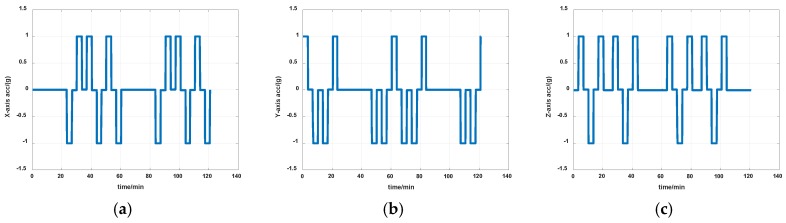
Measurement data of accelerometers in simulation (**a**) *x*-axis; (**b**) *y*-axis; (**c**) *z*-axis.

**Figure 9:**
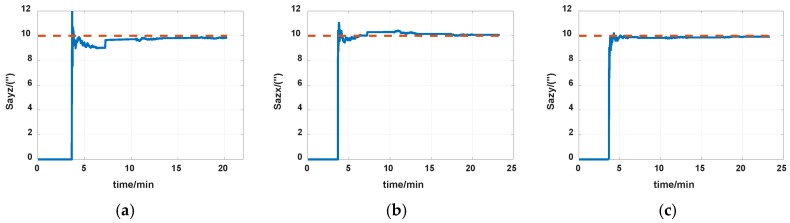
Estimation of accelerometers nonorthogonal installation errors. (**a**) S_ayz_; (**b**) S_azx_; (**c**) S_azy_.

**Figure 10:**
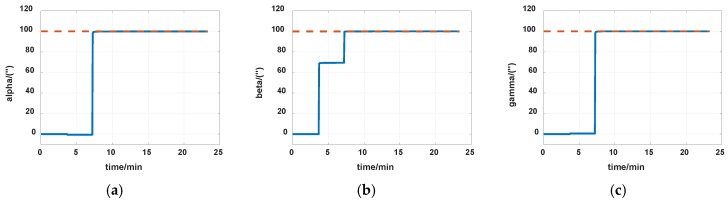
Estimation of installation errors between rotation axes and IMU axes. (**a**) α; (**b**) β; (**c**) γ.

**Figure 11:**
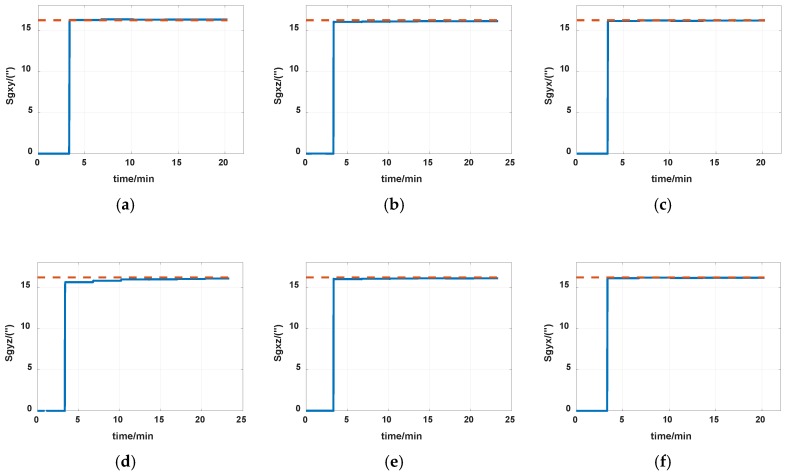
Estimation of gyros nonorthogonal installation errors. (**a**) *S_gxy_*; (**b**) *S_gxz_*; (**c**) *S_gyx_*; (**d**) *S_gyz_*; (**e**) *S_gxz_*; (**f**) *S_gyx_*.

The simulations are first implemented to validate the effectiveness of the proposed self-calibration method. The random error of the gyro was set at 0.005 deg/h, and the random error of the accelerometers was 30 μg, the measurement noise from gyros and accelerometers had the magnitude of 0.2 deg/h and 0.3 μg, respectively. The IMU was in a dynamic process during the calibration, it rotated at an angular rate up to 20 degrees per second, which was a much greater magnitude than the measurement noise. Thus, the measurement noise of IMU can be ignored. The error of the angle of the rotation mechanism was 0.5${}^{\prime \prime }$, while the error of the angular rate of rotation mechanism was 0.7${}^{\prime \prime }/s$. The initial location was 114.19659°E, 30.93041°N. The angular rate of rotation mechanism was set as 20 deg/s (72,000 deg/h). The covariance Q was set as $diag\left(\begin{array}{ccc} w^{2} & \cdots & w^{2} \end{array}\right),w=1^{\prime \prime }$, the dimension of Q was 12, and R in the two Kalman filters were set as $diag\left(\begin{array}{ccc} v_{1}^{2} & v_{1}^{2} & v_{1}^{2} \end{array}\right),\;v_{1}=1\mu g$ and $diag\left(\begin{array}{ccc} v_{2}^{2} & v_{2}^{2} & v_{2}^{2} \end{array}\right),\;v_{2}=1deg/h$, respectively. Measurement data of the IMU in simulation is shown in Figure 7 and Figure 8. The real values of installation errors and their estimation error are shown in Table 1. Figure 9, Figure 10 and Figure 11 describe the comparisons of estimation results of all installation errors, the blue lines represent the estimation of installation errors, and the red dotted lines represent real values.

The time in Figure 9, Figure 10 and Figure 11 was relative, which indicated the convergent tendency of installation errors when the errors were fully activated and could be calibrated. From Table 1 and Figure 9, Figure 10 and Figure 11, we can see all installation errors present good convergence with the proposed method. The estimation errors are all less than 4%.

### 4.2. Experiment

To validate the effectiveness of the proposed method, laboratory experiments were carried out with an actual dual-axis RINS which consists of fiber gyros and quartz accelerometers, whose detailed specifications of dual-axis RINS are shown in Table 2. The covariance Q and R were set to the same as in the simulations. The installation accuracy between the inner axis and outer axis of the dual-axis RINS used in this paper was better than ±4 arc-seconds, thus the inner axis is approximately regarded as perpendicular to the outer axis.

The dual-axis RINS was statically placed on a turntable to isolate external disturbance while the IMU rotates along with the rotation mechanism inside the dual-axis RINS to conduct the calibration.

**Figure 12:**
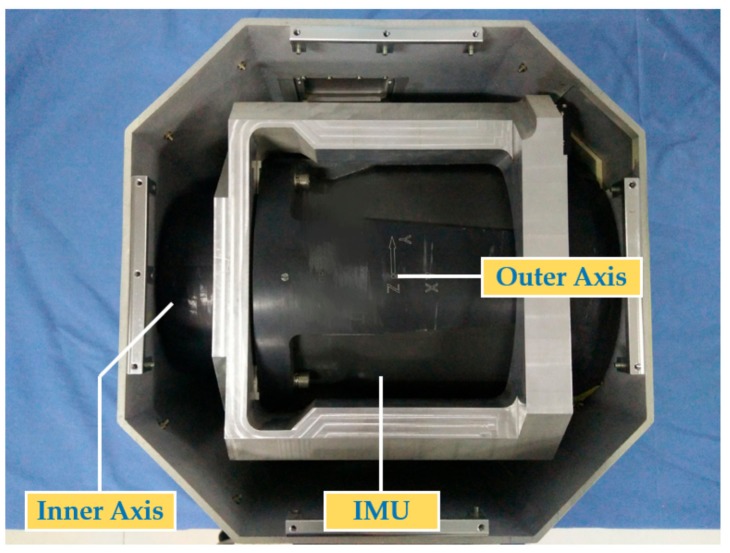
Internal structure of the dual-axis rotational inertial navigation system (RINS).

Considering that the error parameter’s reference cannot be acquired in real situations, estimation results of installation errors using two separating calibration methods mentioned in [11] and [13] were regarded as a reference to prove that the proposed self-calibration method can get comparable accuracy, thereby avoiding the complex separating process. The internal structure of the dual-axis RINS is shown in Figure 12.

**Figure 13:**
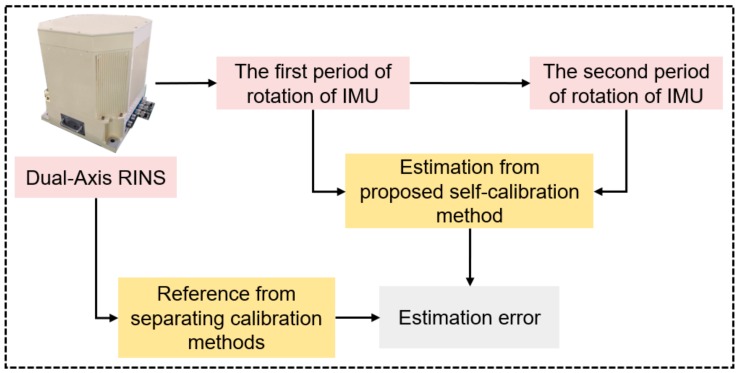
The flow diagram of the experiment.

The experimental procedure is shown in Figure 13. The first period of rotation of the IMU was conducted at first, and Equation (11) was used to estimate nonorthogonal errors of the accelerometers and installation errors between the IMU and rotation axes. Then the results obtained from the first step were sent to the next calibration process. The second period of rotation of the IMU was conducted, and Equation (13) was used to get nonorthogonal errors of gyros. In the end, estimations from the proposed method were compared with counterparts from the separating methods [11,13].

**Figure 14:**
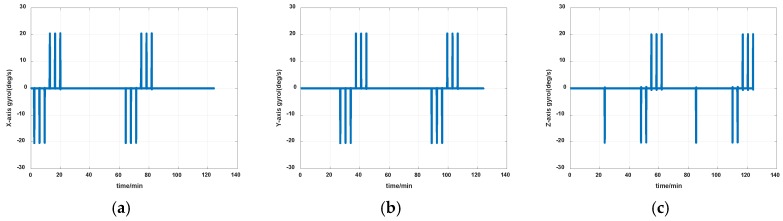
Measurement data of gyros in experiment (**a**) *x*-axis; (**b**) *y*-axis; (**c**) *z*-axis.

**Figure 15:**
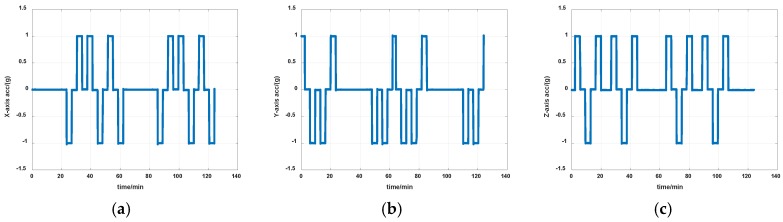
Measurement data of accelerometers in experiment (**a**) *x*-axis; (**b**) *y*-axis; (**c**) *z*-axis.

**Figure 16:**
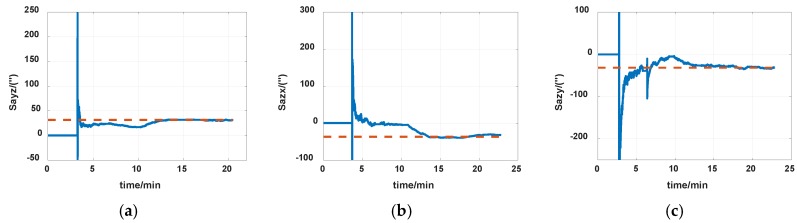
Estimation of accelerometers nonorthogonal installation errors. (**a**) S_ayz_; (**b**) S_azx_; (**c**) S_azy_.

**Figure 17:**
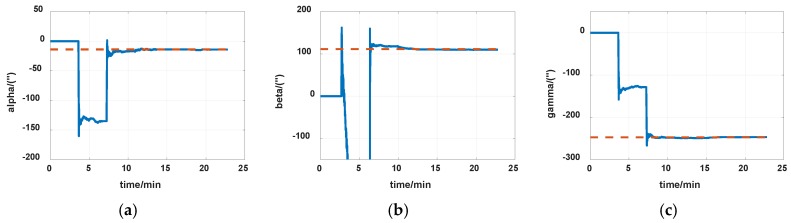
Estimation of installation errors between rotation axes and IMU axes. (**a**) α; (**b**) β; (**c**) γ.

**Figure 18:**
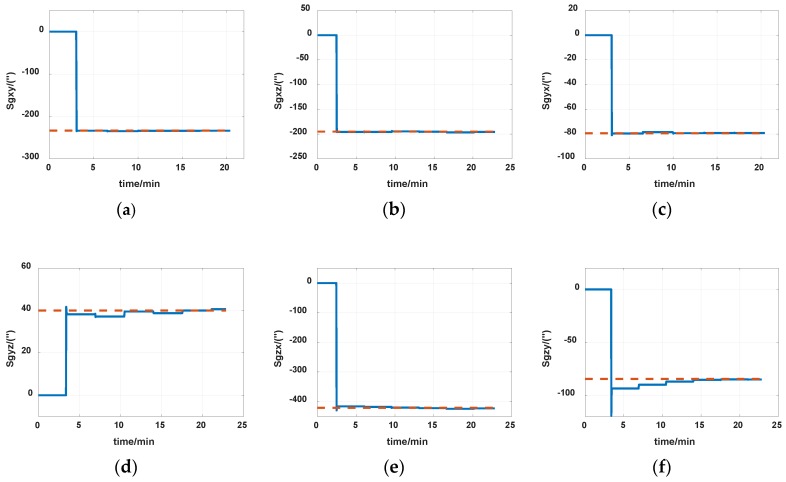
Estimation of gyros nonorthogonal installation errors. (**a**) *S_gxy_*; (**b**) *S_gxz_*; (**c**) *S_gyx_*; (**d**) *S_gyz_*; (**e**) *S_gxz_*; (**f**) *S_gyx_*.

The reference of installation errors and their estimation errors are shown in Table 3. Measurement data of the IMU in the experiment is shown in Figure 14 and Figure 15. Figure 16, Figure 17 and Figure 18 describe the comparisons of the estimation results of all installation errors, the blue lines represent the estimation results of installation errors, and the red line represents the reference.

The time in Figure 16, Figure 17 and Figure 18 was relative, which indicated the convergent tendency of installation errors when the errors were fully activated and could be calibrated. From Table 3 and Figure 16, Figure 17 and Figure 18, we can see that all estimation results present good convergence with the proposed method. All installation errors can be estimated together by the proposed method, which acquires comparable results compared with the separating methods. The estimation errors were all less than 6%.

## 5. Conclusions

A system-level self-calibration method for installation errors of a dual-axis RINS was proposed in this paper. The measurement model was reestablished to ensure that all errors can be estimated together, rather than calibrated separately by utilizing different estimation algorithms and rotation schemes. The relationship between initial IMU attitude and subsequent IMU attitude and angular rate of the rotational mechanism were used to estimate two types of installation errors of RINS, that is, the nonorthogonal installation errors of inertial sensors and installation errors between the IMU axes and rotation axes. The rotation scheme of the IMU was discussed and designed, and the PWCS method was used to analyze the observability of the system. Finally, simulation and laboratory experiments were conducted to validate the proposed method. The results showed that all installation errors of the dual-axis RINS can be accurately estimated by the proposed system-level self-calibration method, thereby avoiding complex separating calibration process.

## Figures and Tables

**Table 1:** Estimation results.

Parameters	Real Values (’’)	Estimation Errors (’’)
$S_{gxy}$	16	0.389
$S_{gxz}$	16	0.575
$S_{gyx}$	16	0.474
$S_{gyz}$	16	0.488
$S_{gzx}$	16	0.544
$S_{gzy}$	16	0.406
$S_{ayz}$	10	0.255
$S_{azx}$	10	0.164
$S_{azy}$	10	0.119
α	100	0.145
β	100	0.042
γ	100	0.067

**Table 2:** Specifications of the dual-axis rotational inertial navigation system (RINS).

Characteristics	Description
Sampling frequency	200 Hz
Gyro bias stability	0.005°/h
Accelerometers bias stability	30 μg
Rotation angle accuracy	0.5${}^{\prime \prime }$
Rotation velocity accuracy	0.7${}^{\prime \prime }/s$

**Table 3:** Estimation results.

Parameters	Real Values (’’)	Estimation Errors (’’)
$S_{gxy}$	−233.615	0.540
$S_{gxz}$	−195.180	1.054
$S_{gyx}$	−79.467	0.573
$S_{gyz}$	39.603	0.698
$S_{gzx}$	−421.075	2.294
$S_{gzy}$	−84.660	0.559
$S_{ayz}$	31.373	0.538
$S_{azx}$	−36.911	1.610
$S_{azy}$	−31.490	1.884
α	−13.828	0.299
β	111.383	1.422
γ	−247.518	1.502
